# Living Alone, Physical Health, and Mortality in Breast Cancer Survivors: A Prospective Observational Cohort Study

**DOI:** 10.3390/healthcare11172379

**Published:** 2023-08-24

**Authors:** Cassie Doyle, Eunjeong Ko, Hector Lemus, Fang-Chi Hsu, John P. Pierce, Tianying Wu

**Affiliations:** 1 Division of Epidemiology and Biostatistics, School of Public Health, San Diego State University, San Diego, CA 92182, USA; cdoyle3871@sdsu.edu (C.D.); hlemus@sdsu.edu (H.L.); 2 School of Social Work, San Diego State University, San Diego, CA 92182, USA; eko@sdsu.edu; 3 Department of Biostatistics and Data Science, Division of Public Health Sciences, Wake Forest University School of Medicine, Winston-Salem, NC 27101, USA; fhsu@wakehealth.edu; 4 Moores Cancer Center, School of Medicine, University of California, San Diego, CA 92037, USA; jppierce@ucsd.edu

**Keywords:** living alone, physical health, mortality, breast cancer survivor, social isolation

## Abstract

Living alone, particularly for individuals with poor physical health, can increase the likelihood of mortality. This study aimed to explore the individual and joint associations of living alone and physical health with overall mortality among breast cancer survivors in the Women’s Healthy Eating and Living (WHEL). We collected baseline, 12-month and 48-month data among 2869 women enrolled in the WHEL cohort. Living alone was assessed as a binary variable (Yes, No), while scores of physical health were measured using the RAND Short Form–36 survey (SF-36), which include four domains (physical function, role limitation, bodily pain, and general health perceptions) and an overall summary score of physical health. Cox proportional hazard models were used to evaluate associations. No significant association between living alone and mortality was observed. However, several physical health measures showed significant associations with mortality (*p*-values < 0.05). For physical function, the multivariable model showed a hazard ratio (HR) of 2.1 (95% CI = 1.02–4.23). Furthermore, the study examined the joint impact of living alone and physical health measures on overall mortality. Among women with better physical function, those living alone had a 3.6-fold higher risk of death (95% CI = 1.01–12.89) compared to those not living alone. Similar trends were observed for pain. However, regarding role limitation, the pattern differed. Breast cancer survivors living alone with worse role limitations had the highest mortality compared to those not living alone but with better role limitations (HR = 2.6, 95% CI = 1.11–5.95). Similar trends were observed for general health perceptions. Our findings highlight that living alone amplifies the risk of mortality among breast cancer survivors within specific health groups.

## 1. Introduction

Breast cancer ranks as the second leading cause of cancer-related mortality among women in the United States [1]. Recent data from January 2022 indicates a population of over 3.8 million female breast cancer survivors in the country [2]. The American Cancer Society defines a breast cancer survivor as any individual who has ever received a cancer diagnosis, irrespective of their current stage of treatment [1].

Survivors of breast cancer have reported a range of physical health challenges during and after their treatment, including fatigue, pain, sleep disturbances, and gastrointestinal issues such as constipation [3]. In addition to these physical ailments, survivors also encounter emotional, psychological, and social concerns. These encompass apprehension regarding cancer metastasis or recurrence, feelings of depression, body image disturbances, changes in interpersonal relationships, and financial burdens [3]. These stressors can adversely impact the well-being and survival of women following a breast cancer diagnosis.

In a comprehensive advisory spanning 85 pages, Surgeon General Vivek Murthy, MD, MBA, released a declaration in May 2023, recognizing loneliness and isolation as significant public health epidemics within the United States [4]. Extensive research has consistently demonstrated a link between social isolation and unfavorable health behaviors, including suboptimal dietary choices, smoking, and physical inactivity [5,6,7]. Furthermore, individuals who experience loneliness and social isolation often face heightened challenges in coping with stress, trauma, adversity, anxiety, and depression [4]. Consequently, breast cancer survivors who also face social isolation are at an increased risk of diminished long-term survival [8,9].

Living alone can contribute to social isolation, as individuals who live by themselves may have limited opportunities for social interaction and companionship. In early 2021, an estimated 37 million adults in the United States aged 18 and older, comprising approximately 15% of the population, were residing alone [10]. These rates of solitary living have been consistently rising and are expected to continue on an upward trajectory [6,10]. While previous studies have investigated social isolation in the context of marital status, social interactions, church group involvement, and community organization membership, less is known specifically about the implications of living alone as an isolated measure among breast cancer survivors [9,11]. However, the existing research on the association between living alone and mortality among various cancer populations has produced limited and mixed findings [6,12,13,14].

Although prior investigations have established a relationship between physical health outcomes and mortality risk in cancer patients [15], no study, to our knowledge, has specifically examined the joint impacts of living alone and physical health on mortality among female breast cancer survivors. This research gap is noteworthy because the influence of living alone on mortality may be contingent upon other factors, such as an individual’s physical health status.

Therefore, the objective of this study is to assess both the independent and combined associations of living alone and physical health with all-cause mortality among female breast cancer survivors in the United States. We will utilize longitudinal data from the Women’s Healthy Eating and Living (WHEL) study to investigate this relationship.

## 2. Materials and Methods

### 2.1. Study Design and Population

This study utilizes data from a pre-existing prospective cohort derived from the WHEL study, which enrolled a total of 3088 female breast cancer survivors between 1995 and 2000, with an average follow-up period of 7.3 years [16]. The WHEL study was a randomized clinical trial supported by the National Institutes of Health (NIH), conducted at seven sites across California, Arizona, Texas, and Oregon [16]). Its primary objective was to investigate the potential benefits of intensive dietary intervention, emphasizing a rich intake of vegetables, fruit, and fiber while minimizing fat consumption, on reducing the incidence of subsequent breast cancer events and premature mortality in women with early-stage invasive breast cancer [16]. Participant recruitment for the study involved a multi-faceted approach, incorporating tumor registries, collaboration with community oncologists, and community outreach initiatives [16]. Detailed information regarding the inclusion and exclusion criteria can be found in the original publication of the WHEL study [16]. In summary, eligible participants were breast cancer survivors aged 18 to 70, with stage I (≥1 cm), II, or IIIA breast cancer diagnosed within the previous four years, not currently scheduled for or undergoing chemotherapy, able to provide dietary data through 24-h food recalls, and exhibiting no evidence of recurrent disease, cancer recurrence, or new breast cancer [16]. Exclusion criteria encompassed pregnancy, the presence of life-threatening diseases or medical conditions, and the diagnosis of comorbidities requiring specific dietary regimens or contraindicating a high-fiber diet due to medication use [16]. Additionally, our study employed supplementary exclusion criteria targeting individuals with missing baseline data on living alone status and physical functioning scores, resulting in a final cohort comprising 2869 women. The data analysis was performed as an observational study rather than a clinical trial, given that the risk factors under examination in this cohort were not the original randomized interventions.

### 2.2. Assessment of Living Alone Status and Physical Health

Living alone status was evaluated as a dichotomous variable, categorizing participants as either living alone or not living alone at three time points: baseline, year 1, and year 4.

Physical health was assessed through the Medical Outcomes Study (MOS) 36-Item Short Form Health Survey (SF-36), a validated instrument developed by RAND in 1992, which specifically measures various aspects of physical health [17]. The SF-36 encompasses four physical health scales, namely physical functioning, role limitations due to physical health problems, bodily pain, and general health perceptions [17]. These scales have been rigorously validated and demonstrate a high level of reliability, with Cronbach’s alpha coefficients ranging from 0.78 to 0.93 [17,18].

The physical functioning scale consists of ten items that assess an individual’s ability to engage in moderate and vigorous physical activities, including tasks such as walking, climbing, bending, and lifting [17]. Role limitations due to physical health are evaluated through four items that examine the extent to which individuals experience limitations in work or other regular activities, such as difficulties in completing tasks or the need for frequent rest [17]. The bodily pain scale comprises two items that measure the degree to which pain interferes with daily activities, encompassing work and household tasks [17]. General health perceptions involve five items that inquire about individuals’ overall assessment of their current health status [17].

Detailed scoring instructions for each scale can be accessed on RAND’s website [19]. Each subscale of physical health is scored on a range from 0 to 100, with higher scores indicating better health. To determine the overall physical health score, the mean of the four physical health subscales was calculated [20].

### 2.3. Assessment of Study Outcome

The primary outcome of this investigation is total mortality. By the conclusion of the study in June 2006, the vital status of 95% of participants in the intervention group and 96% of those in the comparison group was established [21]. Participants who were lost to follow-up were considered censored at the date of their last contact. To determine mortality information, a confirmation interview was conducted with participants, and the medical records and/or death certificates were reviewed independently by two oncologists. Additionally, a search was performed on the National Death Index [16]. The Social Security Death Index (updated until 2009).

Survival analysis was performed by calculating the time from study entry to the occurrence of death. For participants without an event, the follow-up time was censored either at the time of the last documented contact with the study staff or at the completion of the study in June 2006 [16].

### 2.4. Assessments of Covariates

At baseline, standardized questionnaires were employed to gather information on participants’ demographic, behavioral, and lifestyle characteristics. These characteristics, along with self-reported health status, encompassing comorbid conditions (e.g., cardiovascular conditions, digestive conditions, arthritis, osteoporosis) and medications such as blood sugar, cardiovascular, and gastrointestinal medications, were documented [16]. Relevant variables pertaining to the patient’s medical records were also extracted, including their initial cancer diagnosis and treatment details [16]. Variables concerning cancer status and treatment encompassed hormone receptor status and the utilization of radiation or chemotherapy [16].

To evaluate physical activity levels, an adapted and validated Personal Habits Questionnaire from the Women’s Health Initiative was employed [22]. Physical activity was quantified using metabolic equivalent tasks (METs), as employed in prior studies [23]. Additionally, social support was evaluated utilizing the 9-item MOS social support scale, which exhibited good internal consistency with a Cronbach’s alpha of 0.75 [17].

### 2.5. Statistical Analyses

All data analyses were performed using SAS version 9.4 (SAS Institute, Cary, NC, USA). To assess the differences in baseline characteristics, we employed the χ^2^ test for categorical variables and *t*-test and analysis of variance (ANOVA) for normally distributed continuous variables for both (a) living alone status, physical function score, role limitation score, pain score, general health score, and overall physical health score and (b) mortality status.

Living alone status was treated as a binary variable (Yes, No), while the physical function summary score (≤Median, >Median) and all other physical health scores (<Median, ≥Median) were categorized into two groups for ease of interpretation. Repeated measures of living alone status and physical health scores at years 0, 1, and 4 were analyzed as time-varying covariates.

Cox proportional hazard models were utilized to examine the independent and joint associations of living alone and each subscale of physical health with all-cause mortality. Hazard ratios (HRs) and their corresponding 95% confidence intervals (CIs) were used to estimate these associations. We examined the joint associations by creating joint variables of living alone with each subscale of physical health. Each joint variable consisted of 4 groups of living arrangements and each individual physical health subscale. We first divided each physical health subscale into two categories using the median score as a cut-point. Then, we created the 4 joint groups to represent the interaction of physical function and living arrangements. The four groups include Physical function score > median combined with not living alone, physical function score > median combined with living alone, physical function score ≤ median combined with not living alone, and physical function score ≤ median combined with living alone. Analogous procedures were employed to generate all remaining joint variables. We included relevant covariates in our models.

Adjustments were made for the following covariates based on a priori assumptions: age at diagnosis (years), cancer stage (I, II, IIIA), chemotherapy (Yes, No), radiotherapy (Yes, No), hormone status (ER+/PR+, Other), ethnicity (White, Non-white), social support summary score (2–42, 42–67, 67–89, 89–100), body mass index (BMI) in kg/m^2^ (Underweight, Healthy weight, Overweight, Obese), alcohol consumption in g (0, ≤0.14, 0.14–5.95, 5.95–16.17, >16.17), physical activity in METs/week (≤225, 225–675, 675–1350, and >1350), smoking status (Never smoker, Past smoker with less than 15 pack years, Past smoker with 15 or more pack years, Current smoker), menopausal status (Premenopausal, Postmenopausal, Perimenopausal), and medical conditions requiring medications (None, 1, 2, 3 or more). Time-varying covariates included the social support summary score, BMI, alcohol intake, physical activity, and smoking status. Baseline data were used for the other covariates. As the main study was a randomized trial, we included group assignment (Intervention, Comparison) in the model to account for the possibility of intervention effects on other variables. The proportional hazards assumption was assessed and met for all Cox proportional hazard regression models.

## 3. Results

### 3.1. Baseline Characteristics

Table 1 presents the baseline characteristics of the 2869 women included in the study. Most participants were White (85.6%), with a mean age of 50.8 years at the time of diagnosis. A large proportion of these women did not live alone (83.8%) and were postmenopausal (79.7%). Approximately 56.3% of the participants had physical function scores equal to or lower than the median score of 90. Furthermore, a considerable number of women underwent chemotherapy (69.7%), and radiotherapy (61.4%), and 58.2% had no medical conditions requiring medications. Among the participants, 62.0% were classified as ER+/PR+, and 54.1% were never smokers. Nearly half of the participants had a healthy weight (42.1%), and approximately 31.4% reported no alcohol intake.

Throughout the follow-up period, a total of 288 deaths occurred. Comparing women who experienced all-cause mortality to those without any deaths, we observed higher rates of living alone (21.2%), physical functioning scores below the median (66.7%), obesity based on BMI (33.3%), a history of smoking with 15 or more pack years (22.2%), and a higher likelihood of belonging to the lower two groups of physical activity, reporting less than 675 METs of physical activity per week (61.4%). The *p*-values for these comparisons were <0.05.

### 3.2. Baseline Characteristics Stratified by Physical Function Score and Living Arrangement

Table 2 presents the unadjusted bivariate associations between baseline characteristics and physical function scores, as well as living alone status. In comparison to women with better physical health based on their function scores, women with poorer health were more likely to be older, overweight, or obese, have lower social support scores (falling into the two lowest categories), report lower levels of physical activity (falling into the two lowest categories), and have one, two, or three or more medical conditions requiring medications. As indicated in Table 2, women who lived alone, in contrast to those who did not, were more likely to be older and have lower social support scores (falling into the three lowest categories). Additionally, women who lived alone were less likely to have never smoked and to have undergone chemotherapy. The *p*-values for these comparisons were <0.05.

### 3.3. Independent Associations of Living Alone, Physical Function, and Other Physical Health Scores with Mortality

The associations of living alone, physical function, role limitation, pain, general health, and overall physical health with all-cause mortality are presented in Table 3. Age-adjusted and multivariable-adjusted analyses were conducted to assess these associations.

No significant association between living alone and all-cause mortality was found. The age-adjusted hazard ratio (HR) for living alone was 1.3 (95% CI = 0.72–2.53), while the multivariable-adjusted HR was 1.4 (95% CI = 0.75–2.78).

A significant association was observed between physical function and all-cause mortality (*p*-value < 0.05). In the age-adjusted model, women with lower physical function had a higher risk of death, with an HR of 2.7 (95% CI = 1.36–5.19). After adjusting for covariates in the multivariable model, the association remained significant but slightly attenuated, with an HR of 2.1 (95% CI = 1.02–4.23). Role limitation due to physical health issues was also significantly associated with all-cause mortality (*p*-value < 0.05). The age-adjusted HR for role limitation was 2.0 (95% CI = 1.16–3.59), and the multivariable-adjusted HR was 1.8 (95% CI = 1.03–3.32).

No significant association was found between pain and mortality. However, general health showed a significant association with all-cause mortality (*p*-value < 0.05), with an age-adjusted HR of 2.6 (95% CI = 1.47–4.56) and a multivariable-adjusted HR of 2.6 (95% CI = 1.41–4.67).

Regarding overall physical health, a significant association with mortality was observed in the age-adjusted model, with an HR of 2.5 (95% CI = 1.20–5.10). However, in the multivariable-adjusted model, the association was not statistically significant, with an HR of 1.9 (95% CI = 0.86–4.03).

### 3.4. Joint Associations of Physical Health and Living Alone with Mortality

**Figure 1:**
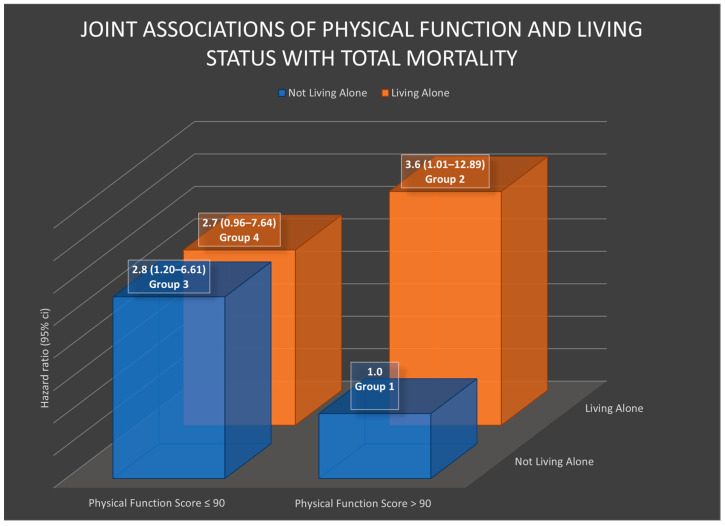
The hazard ratios in this chart were adjusted for intervention group, age at diagnosis, body mass index, cancer stage, estrogen and progesterone status, alcohol consumption, physical activity levels, social support, smoke years, radiotherapy, chemotherapy, menopausal status, and number of comorbidity medications.

**Figure 2:**
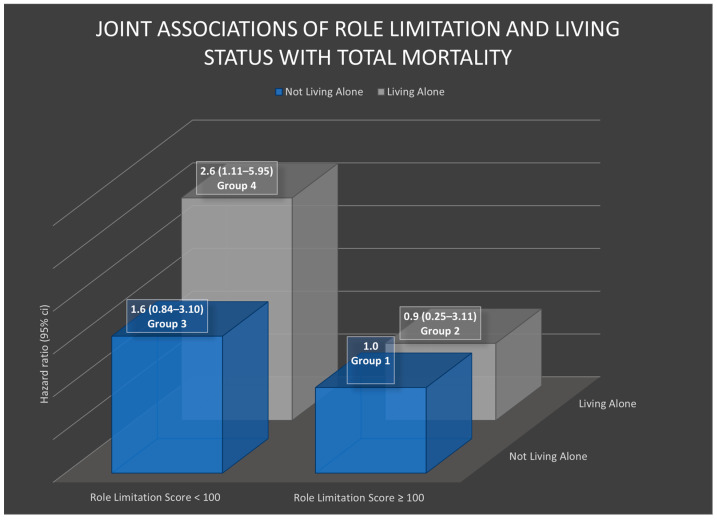
The hazard ratios in this chart were adjusted for intervention group, age at diagnosis, body mass index, cancer stage, estrogen and progesterone status, alcohol consumption, physical activity levels, social support, smoke years, radiotherapy, chemotherapy, menopausal status, and number of comorbidity medications.

The joint impacts of living alone and each subscale of physical health on all-cause mortality were examined. Table 4, Figure 1 and Figure 2 present the results of these joint associations.

As seen in Table 4 and Figure 1, among women with higher physical function, those who were living alone showed a significantly higher rate of death compared to women who were not living alone and had higher physical function scores (*p*-value < 0.05). The hazard ratio (HR) was 3.6 (95% CI = 1.01–12.98), indicating that women living alone with higher physical function had 3.6 times the risk of mortality compared to the reference group. In contrast, for individuals with lower physical function scores, the HRs were similar between women who lived alone and those who did not live alone, with HRs ranging from 2.7 to 2.8 and significant or marginally significant confidence intervals.

Furthermore, the joint associations of living alone with role limitation, pain, general health, and overall physical health on all-cause mortality were examined, as presented in Table 4. Similar trends were observed for the joint associations of pain with living alone, resembling the joint associations of physical function with living alone. However, for role limitation and general health, the trends of the joint associations differed somewhat. As seen in Table 4 and Figure 2, Group 4, which consisted of individuals living alone with lower levels of role limitation or general health, exhibited the highest mortality compared to the reference group (Group 1), with hazard ratios of 2.6 (95% CI = 1.11–5.95) for role limitation and 4.0 (95% CI = 1.66–9.82) for general health.

In contrast to other measures, the joint analysis of living alone and overall physical health revealed that living alone did not further increase the risk of mortality in either the groups with lower or higher scores of overall physical health.

Overall, these findings highlight the complex interplay between living alone and various aspects of physical health in relation to all-cause mortality.

## 4. Discussion

The results of our study revealed significant associations between several measures of physical health and an increased risk of total mortality. While living alone showed a positive association with total mortality, the independent association did not reach statistical significance. Additionally, we observed the joint associations between living alone and various physical measures in relation to mortality.

Regarding role limitation and general health, individuals who lived alone and had lower levels of role limitation or general health exhibited a greater risk of mortality compared to those who also lived alone but had higher levels of role limitation or better general health. Conversely, for physical function and pain, living alone significantly increased the risk of mortality among individuals with better physical function or better levels of pain. However, for these two measures, living alone did not further amplify the risk of mortality among individuals with worse levels of physical function or worse levels of pain.

Our finding regarding the significant association between physical health and higher mortality rates aligns with previous studies [8,15,24,25]. However, the association between living alone and total mortality did not reach statistical significance, possibly due to the relatively shorter follow-up time of 6 years in our study. The lack of a significant association between living alone and higher mortality rates has been reported in some studies, indicating that living alone is not the sole predictor of mortality in general and cancer-specific populations [6,12,13,14]. The estimated hazard ratio for the association between living alone and mortality in our study falls within the range of estimates reported by prior studies, which ranged from 0.93 to 1.81 [6,13,14].

It should be noted that living alone does not necessarily lead to mortality. However, it may contribute to individuals receiving limited instrumental support [9], which could explain why living alone, in conjunction with poor physical health, accelerates the risk of mortality, particularly concerning role limitation and general health measures. The reasons underlying the increased mortality risk associated with living alone in the high physical function and lower pain group but not in the low physical function or high pain group remain unclear. Nonetheless, it is essential to recognize that lower physical function itself is significantly associated with mortality, suggesting that the impact of living alone on mortality may be relatively small compared to the effect of physical function. This could elucidate why significant associations were not observed in the latter group, although the presence of measurement errors or residual confounding cannot be entirely ruled out. Nevertheless, our findings highlight that living alone amplifies the risk of mortality within specific physical health groups.

This analysis possesses several strengths. Notably, it employed a joint modeling approach to assess the simultaneous associations of living alone, physical functioning, and additional physical health measures with mortality. We utilized validated and standardized scales to measure quality of life, and serial measurements were employed to capture living alone and physical function over time. Moreover, our study encompassed a substantial sample size of women with early-stage breast cancer, with a follow-up period of 7.3 years, affording adequate statistical power to identify associations with mortality and control for potential confounding variables, including demographic and lifestyle factors. Furthermore, reported deaths were verified through death certificates, and vital status was confirmed using the National Death Registry.

A limitation of our study is that we utilized a single, well-validated, yet questionnaire-based measure (SF-36) instead of employing multiple objective measures. The reliance on self-reported responses for these measures introduces the potential for measurement errors influenced by bias. Although socioeconomic status adjustment was not feasible, we did control for social support within our study. Additionally, our sample predominantly consisted of White breast cancer survivors, limiting the generalizability of our findings to other demographic and general populations. A notable limitation of our study is related to the temporal scope of the data utilized in our study. The dataset stems from the WHEL study conducted between 1995 and 2000, prompting concerns regarding its applicability to the current context in 2023. It’s reasonable to acknowledge that the social landscape and lifestyle patterns have likely evolved over the past two decades. Despite this, we assert that certain fundamental physiological processes influenced by living alone and physical health remain the same. For instance, the well-established connection between physical health and mortality is less prone to drastic alterations over time. In addition, it’s crucial to bring attention to the prevailing trend of increased prevalence in individuals living alone, as evidenced by refs. [6,10]. This noteworthy rise in solitary living defies assumptions and emphasizes the ongoing relevance and importance of our research topic. In this contemporary context, the physiological and health implications of living alone might hold even greater significance, given the heightened prevalence of this living arrangement. Regarding the temporal extent of data collection, it’s worth noting that while the baseline data was collected between 1995 and 2000, the study encompassed a follow-up period extending until 2006, and the death index was updated until 2009. This extended data collection duration affords us a broader range of information over time, potentially offering insights into the dynamics and changes that occurred during this period.

We acknowledge that our findings may serve as preliminary data, laying the groundwork for future research endeavors. As such, we encourage future studies to build upon our work, ideally utilizing more recent and comprehensive datasets that encompass a broader spectrum of variables and an extended follow-up period. Given the current lack of consensus on the concept of social isolation in cancer populations, it is recommended that further research be conducted to consider the development and implementation of new social isolation assessment tools that encompass both subjective and objective measures of social isolation [26,27]. Furthermore, we suggest implementing community-based interventions that foster social connections and physical activity. These interventions may involve initiatives like revitalizing public spaces or establishing community exercise programs, as supported by previous studies [4,28,29,30,31].

## 5. Conclusions

This study highlights the significant joint impact of living alone and physical health on the mortality of breast cancer survivors, emphasizing the importance of addressing this social issue—living alone. Healthcare professionals should be aware of the living arrangements of cancer survivor patients, particularly if they are living alone. It is crucial for healthcare professionals, including nurses and physicians, to utilize social isolation assessment tools that are specifically designed for cancer survivors. This approach is vital in delivering precise and tailored care to meet the unique needs of these patients. Moreover, with a mindful acknowledgment of the unique health effects tied to living alone, healthcare administrators and policymakers can devise approaches fostering increased opportunities for community socialization and physical activity. This might entail enhancing access to green and public spaces, especially in urban marginalized communities prone to experiencing collective isolation, which could decrease the mortality risk from breast cancer [31,32,33].

## Figures and Tables

**Table 1:** Baseline characteristics of the WHEL study participants (*n* = 2869).

	Overall	Mortality Status		*p*-Value
		No (*n* = 2581)	Yes (*n* = 288)	
Live alone				**0.016**
No	2404 (83.8)	2177 (84.4)	227 (78.8)	
Yes	465 (16.2)	404 (15.7)	61 (21.2)	
Physical function score				**0.0002**
≤90	1615 (56.3)	1423 (55.1)	192 (66.7)	
>90	1254 (43.7)	1158 (44.8)	96 (33.3)	
Role limitation score				**<0.0001**
<100	1317 (45.9)	1150 (44.6)	167 (58.0)	
≥100	1550 (54.0)	1429 (55.4)	121 (42.0)	
Pain score				
<87.5	1433 (50.0)	1270 (49.2)	163 (56.6)	**0.0027**
≥87.5	1435 (50.0)	1310 (50.8)	125 (43.4)	
General health score				**0.0025**
<75	1322 (46.1)	1165 (45.1)	157 (54.5)	
≥75	1547 (53.9)	1416 (54.9)	131 (45.5)	
Overall physical health score				**0.034**
<43.125	2090 (72.9)	1865 (72.3)	225 (78.1)	
≥43.125	779 (27.2)	716 (27.7)	63 (21.9)	
Age at diagnosis (years)	50.8 ± 8.8	50.8 ± 8.7	51.4 ± 10.1	0.24
Randomization group				0.78
Intervention	1427 (49.7)	1286 (49.8)	141 (49.0)	
Comparison	1442 (50.3)	1295 (50.2)	147 (51.0)	
Cancer Stage				**<0.0001**
I	1107 (38.6)	1054 (40.8)	53 (18.4)	
II	1618 (56.4)	1422 (55.1)	196 (68.1)	
IIIA	144 (5.0)	105 (4.1)	39 (13.5)	
Chemotherapy				**0.0016**
Yes	1997 (69.7)	1773 (68.8)	224 (77.8)	
No	870 (30.4)	806 (31.3)	64 (22.2)	
Radiation therapy				0.89
Yes	1760 (61.4)	1582 (61.34)	178 (61.8)	
No	1105 (38.6)	995 (38.6)	110 (38.2)	
Hormone status				**<0.0001**
ER+/PR+	1778 (62.0)	1631 (63.2)	147 (51.0)	
Other	1091 (38.0)	950 (36.8)	141 (49.0)	
Ethnicity				0.18
White	2457 (85.6)	2218 (85.9)	239 (83.0)	
Non-white	412 (14.4)	363 (14.1)	49 (17.0)	
Social support summary score (quartile)				0.50
2–42	142 (5.0)	122 (4.7)	20 (6.9)	
42–67	522 (18.2)	475 (18.4)	47 (16.3)	
67–89	1126 (39.3)	1015 (39.3)	111 (38.5)	
89–100	1078 (37.6)	968 (37.5)	110 (38.2)	
BMI (kg/m^2^)				**0.0063**
Underweight (0–18.5)	28 (1.0)	23 (0.9)	5 (1.7)	
Healthy weight (18.5–25)	1209 (42.1)	1108 (42.9)	101 (35.1)	
Overweight (25–30)	885 (30.9)	799 (31.0)	86 (29.9)	
Obese (≥30)	747 (26.0)	651 (25.2)	96 (33.3)	
Alcohol consumption (g)				0.13
0	902 (31.4)	799 (31.0)	103 (35.8)	
≤0.14	492 (17.2)	434 (16.8)	58 (20.1)	
0.14–5.95	709 (24.7)	646 (25.0)	63 (21.9)	
5.95–16.17	448 (15.6)	410 (15.9)	38 (13.2)	
>16.17	318 (11.1)	292 (11.3)	26 (9.0)	
Physical activity (METs/week)				**0.0072**
≤225	805 (28.1)	713 (27.6)	92 (31.9)	
225–675	710 (24.8)	625 (24.2)	85 (29.5)	
675–1350	715 (24.9)	648 (25.1)	67 (23.3)	
>1350	639 (22.3)	595 (23.1)	44 (15.3)	
Smoking status				**<0.0001**
Never smoker	1553 (54.1)	1409 (54.6)	144 (50.0)	
Past smoker with less than 15 pack years	750 (26.1)	692 (26.8)	58 (20.1)	
Past smoker with 15 or more pack years	421 (14.7)	357 (13.8)	64 (22.2)	
Current smoker	126 (4.4)	110 (4.3)	16 (5.6)	
Menopausal status				**0.049**
Premenopausal	313 (10.9)	271 (10.5)	42 (14.6)	
Postmenopausal	2283 (79.7)	2057 (79.8)	226 (78.5)	
Perimenopausal	269 (9.4)	249 (9.7)	20 (6.9)	
Tumor Size (centimeters)				**<0.0001**
≤2	1667 (58.3)	1563 (60.8)	104 (36.1)	
>2	1194 (41.7)	1010 (39.3)	184 (63.9)	
Medical conditions that require medications				**0.036**
None	1670 (58.2)	1507 (58.4)	163 (56.6)	
1	691 (24.1)	615 (23.8)	76 (26.4)	
2	349 (12.2)	324 (12.6)	25 (8.7)	
3 or more	159 (5.5)	135 (5.2)	24 (8.3)	

Categorical variables are presented as N, % (column %). Continuous variables are presented as mean ± SD. Abbreviations: ER = estrogen receptor; PR = progesterone receptor; BMI = body mass index; METs = metabolic equivalents. Bolded *p*-Values indicate statistical significance using a significance level (α) of 0.05.

**Table 2:** Bivariate associations of baseline characteristics with physical function scores and living arrangements among breast cancer survivors (*n =* 2869).

	Physical Function Score			Live Alone		
	>90	≤90	*p*-Value	No	Yes	*p*-Value
Age at diagnosis (years)	49.2 ± 8.8	52.1 ± 8.7	**<0.0001**	50.3 ± 8.8	53.7 ± 8.7	**<0.0001**
Randomization group			0.96			0.46
Intervention	623 (49.7)	804 (49.8)		1203 (50.0)	224 (48.2)	
Comparison	631 (50.3)	811 (50.2)		1201 (50.0)	241 (51.8)	
Cancer Stage			0.081			0.95
I	492 (39.2)	615 (38.1)		927 (38.6)	180 (38.7)	
II	712 (56.8)	906 (56.1)		1355 (56.4)	263 (56.6)	
IIIA	50 (4.0)	94 (5.8)		122 (5.1)	22 (4.7)	
Chemotherapy			0.48			**<0.0001**
Yes	882 (70.3)	1115 (69.1)		1718 (71.5)	279 (60.0)	
No	372 (29.7)	498 (30.9)		684 (28.5)	186 (40.0)	
Radiation therapy			0.85			0.60
Yes	771 (61.6)	989 (61.3)		1480 (61.6)	280 (60.3)	
No	480 (38.4)	625 (38.7)		921 (38.4)	184 (39.7)	
Hormone status			0.49			0.77
ER+/PR+	786 (62.7)	992 (61.4)		1487 (61.9)	291 (62.6)	
Other	468 (37.3)	623 (38.6)		917 (38.1)	174 (37.4)	
Social support summary score (Quartile)			**<0.0001**			**<0.0001**
2–42	37 (2.9)	105 (6.5)		86 (3.6)	56 (12.0)	
42–67	179 (14.2)	350 (21.5)		380 (15.7)	149 (32.0)	
67–89	498 (39.5)	635 (39.0)		949 (39.2)	184 (39.5)	
89–100	547 (43.4)	537 (33.0)		1007 (41.6)	77 (16.5)	
BMI (kg/m^2^)			**<0.0001**			**0.046**
Underweight (0–18.5)	14 (1.1)	14 (0.9)		19 (0.8)	9 (1.9)	
Healthy weight (18.5–25)	695 (55.1)	514 (31.6)		1000 (41.3)	209 (44.9)	
Overweight (25–30)	378 (30.0)	507 (31.1)		754 (31.1)	131 (28.1)	
Obese (≥30)	167 (13.2)	580 (35.6)		631 (26.0)	116 (24.9)	
Alcohol consumption (g)			**<0.0001**			0.18
0	354 (28.1)	548 (33.7)		746 (30.8)	156 (33.5)	
≤0.14	183 (14.5)	329 (20.2)		432 (17.8)	80 (17.2)	
0.14–5.95	308 (24.4)	401 (24.6)		612 (25.3)	97 (20.8)	
5.95–16.17	236 (18.7)	212 (13.0)		376 (15.5)	72 (15.5)	
>16.17	180 (14.3)	138 (8.5)		257 (10.6)	61 (13.1)	
Physical activity (METs/week)			**<0.0001**			0.21
0–225	215 (17.1)	590 (36.2)		690 (28.5)	115 (24.7)	
225–675	286 (22.7)	424 (26.0)		587 (24.2)	123 (26.4)	
675–1350	349 (27.7)	366 (22.5)		589 (24.3)	126 (27.0)	
>1350	404 (32.0)	235 (14.4)		538 (22.2)	101 (21.7)	
Smoking status			0.15			**<0.0001**
Never smoker	693 (55.0)	860 (52.8)		1355 (55.9)	198 (42.5)	
Past smoker with less than 15 pack years	338 (26.8)	412 (25.3)		618 (25.5)	132 (28.3)	
Past smoker with 15 or more pack years	162 (12.9)	259 (15.9)		320 (13.2)	101 (21.7)	
Current smoker	54 (4.3)	72 (4.4)		95 (3.9)	31 (6.7)	
Menopausal status			**<0.0001**			**<0.0001**
Premenopausal	191 (15.3)	122 (7.6)		288 (12.0)	25 (5.4)	
Postmenopausal	951 (76.0)	1332 (82.6)		1884 (78.4)	399 (86.2)	
Medical conditions that require medications			**<0.0001**			0.15
None	875 (69.8)	795 (49.2)		1421 (59.1)	249 (53.6)	
1	267 (21.3)	424 (26.3)		569 (23.7)	122 (26.2)	
2	91 (7.3)	258 (16.0)		283 (11.8)	66 (14.2)	
3 or more	21 (1.7)	138 (8.5)		131 (5.5)	28 (6.0)	

Categorical variables are presented as N, % (column %). Continuous variables are presented as mean ± SD. Abbreviations: ER = estrogen receptor; PR = progesterone receptor; BMI = body mass index; METs = metabolic equivalents. Bolded *p*-Values indicate significance using a statistical significance level (α) of 0.05.

**Table 3:** Independent associations of living alone and each physical health measure with total mortality.

	Category	Age-Adjusted HR (95% CI)	Multivariable-Adjusted HR (95% CI)
Living alone	No	Ref	Ref
	Yes	1.3 (0.72–2.53)	1.4 (0.75–2.78)
Physical function	>90	Ref	Ref
	≤90	2.7 (1.36–5.19) *	2.1 (1.02–4.23) *
Role limitation	≥100	Ref	Ref
	<100	2.0 (1.16–3.59) *	1.8 (1.03–3.32) *
Pain	≥87.5	Ref	Ref
	<87.5	1.3 (0.77–2.28)	1.1 (0.63–1.97)
General health	≥75	Ref	Ref
	<75	2.6 (1.47–4.56) *	2.6 (1.41–4.67) *
Overall physical health	≥43.125	Ref	Ref
	<43.125	2.5 (1.20–5.10) *	1.9 (0.86–4.03)

* *p*-value < 0.05. The multivariable-adjusted model was adjusted for intervention group, age at diagnosis, BMI, cancer stage, estrogen and progesterone status, alcohol consumption, physical activity levels, social support, smoking status, radiotherapy, chemotherapy, menopausal status, and number of comorbidity medications.

**Table 4:** Joint associations of living alone and each physical health measure with mortality.

	Living Alone	
	No HR (95% CI)	Yes HR (95% CI)
Physical function		
>90	Ref	3.6 (1.01–12.89) *
≤90	2.8 (1.20–6.61) *	2.7 (0.96–7.64)
Role limitation		
≥100	Ref	0.9 (0.25–3.11)
<100	1.6 (0.84–3.10)	2.6 (1.11–5.95) *
Pain		
≥87.5	Ref	2.6 (1.05–6.55) *
<87.5	1.5 (0.78–2.90)	1.2 (0.45–3.14)
General health		
≥75	Ref	1.0 (0.33–3.24)
<75	2.3 (1.16–4.48) *	4.0 (1.66–9.82) *
Overall physical health		
≥43.125	Ref	1.6 (0.78–3.09)
<43.125	2.2 (0.97–5.10)	1.1 (0.14–8.60)

* *p*-value < 0.05. The multivariable-adjusted model was adjusted for intervention group, age at diagnosis, BMI, cancer stage, estrogen and progesterone status, alcohol consumption, physical activity levels, social support, smoke years, radiotherapy, chemotherapy, menopausal status, and number of comorbidity medications.

## Data Availability

Most of the data used in this paper can be found at: https://library.ucsd.edu/dc/object/bb2493244b (accessed on 18 April 2022).
